# Making music for mental health: how group drumming mediates recovery

**DOI:** 10.1186/s13612-016-0048-0

**Published:** 2016-11-29

**Authors:** Rosie Perkins, Sara Ascenso, Louise Atkins, Daisy Fancourt, Aaron Williamon

**Affiliations:** 1 Centre for Performance Science, Royal College of Music, Prince Consort Road, London, SW7 2BS UK; 2 Faculty of Medicine, Imperial College London, London, SW7 2AZ UK

**Keywords:** Music, Group drumming, Recovery mechanisms, Mental health, Qualitative

## Abstract

**Background:**

While music-making interventions are increasingly recognised as enhancing mental health, little is known of why music may engender such benefit. The objective of this article is to elucidate the features of a programme of group drumming known to enable mental health recovery.

**Methods:**

Qualitative research was conducted with 39 mental health patients and carers who had demonstrated recovery following engagement with a programme of group djembe drumming in the UK. Data were collected through semi-structured individual interviews and focus group interviews designed to understand the connection between drumming and recovery and analysed using Interpretative Phenomenological Analysis (IPA).

**Results:**

Results revealed three overarching features of the drumming intervention: (1) the specific features of drumming, including drumming as a form of non-verbal communication, as a connection with life through rhythm, and as a grounding experience that both generates and liberates energy; (2) the specific features of the group, including the group as a space of connection in and through the rhythmic features of the drumming, as well as facilitating feelings of belonging, acceptance, safety and care, and new social interactions; (3) the specific features of the learning, including learning as an inclusive activity in which the concept of mistakes is dissolved and in which there is musical freedom, supported by an embodied learning process expedited by the musical facilitator.

**Conclusion:**

The findings provide support for the conceptual notion of ‘creative practice as mutual recovery’, demonstrating that group drumming provides a creative and mutual learning space in which mental health recovery can take place.

**Electronic supplementary material:**

The online version of this article (doi:10.1186/s13612-016-0048-0) contains supplementary material, which is available to authorized users.

## Background

Mental healthcare in the UK remains a challenge, in terms of large numbers of people suffering from mental distress and perceived shortcomings in treatment and care (Crawford et al. 2013; Royal Society for Public Health (RSPH) 2012). The extent of mental disorders—estimated at 38.2% of the EU population (Wittchen et al. 2011)—means that there is considerable incentive to enhance mental health. Building upon the World Health Organization’s argument that ‘mental health implies fitness rather than freedom from illness’ (WHO 2004, p. 14), mental health is increasingly viewed in terms of *positive health*: ‘a state beyond the mere absence of disease [which] is definable and measurable’ (Seligman 2008, p. 3). To think of mental health in this way means to focus attention not on symptoms but rather on ways in which we can *be well,* or in other words on *wellbeing.* This article explores the relationships between music-making and wellbeing, focusing particularly on *why* music may be able to optimise positive mental health.

According to Seligman (2011), wellbeing is sustained by five elements: Positive emotions (including happiness and life satisfaction), Engagement (complete immersion in an activity), Relationships (being cared for and valued), Meaning (significance of life and belonging to something larger than the self), and Accomplishment (achievement and mastery). These PERMA elements focus on the ways in which we can be well, rather than on symptoms of ill health, and include both hedonic (feeling good) and eudaimonic (functioning well) aspects of wellbeing. Similarly, Keyes (2007 p. 98) makes an argument that mental health should be considered in terms of what he terms ‘flourishing’, clustered around the presence of three factors: positive emotions, positive psychological functioning, and positive social functioning. Drawing on these conceptual starting points, mental health is framed in what follows as optimal, multidimensional wellbeing rather than symptoms of illbeing.

Aligned to this way of thinking, Crawford et al. (2013) write of the notion of *recovery*: ‘the possibility of achieving a meaningful and more resilient life irrespective of mental health “symptoms” or disabilities’ (p. 55). Recovery can be viewed as leading a fulfilling life as defined by the individual, building a life beyond illness without necessarily eliminating the symptoms of illness, and can be characterised as a journey of being *in* recovery rather than recovered (South London and Maudsley National Health Service Foundation Trust and South West London and St George’s Mental Health National Health Service Trust 2010, p. 4). Importantly, the concept of recovery empowers service users (Amering and Schmolke 2009), but it can also be extended to include those who care—informally or formally—for others. It is the notion of mental health recovery, as a process in which individuals can achieve a meaningful and more resilient life regardless of mental health symptoms, which this article explores. In particular, it scrutinises the role that music-making may play as a mediator of recovery.

### Music as a mediator of recovery

Crawford et al. (2013) propose that the arts may enable new ways of enabling recovery in community contexts, providing forums of compassion, trust, and shared understanding in which people can find the opportunity to express and understand their experiences and rebuild identities. The relatively new field of *Health humanities* (Crawford et al. 2015) lends support to this idea, based upon the notion that ‘arts and humanities [are] a core constituent and enabler of health and well-being by transforming places, processes and people, whether in hospitals, clinics, schools, prisons or community settings’ (p. 19). Certainly, the field of arts and health is now well established (Bungay et al. 2014; Staricoff 2006; The RSPH Working Group on Arts, Health and Wellbeing 2013), and there is growing evidence that music is an enabler of mental health recovery among diverse populations (see MacDonald et al. 2012a).

Listening to music, for example, has been established as a means of maintaining wellbeing among older adults (Hays and Minichiello 2005; Laukka 2007). For this same population, there is also evidence to suggest that engaging in active musical activity (e.g. singing or playing an instrument) can contribute to demonstrable improvements in factors such as subjective wellbeing (Creech et al. 2013), anxiety levels (Hars et al. 2014), depression and mood (Seinfeld et al. 2013), and morale and loneliness (Cohen et al. 2006). Looking at drumming in particular, this form of music-making has been shown to facilitate recovery among vulnerable populations such as young people (Faulkner et al. 2012; Wood et al. 2013), social workers (Maschi et al. 2013), sex workers in a rehabilitation programme (Venkit et al. 2013), and mental health service users (Fancourt et al. 2016a, 2016b). Aligned with many of the principles of recovery, some of the documented benefits from music engagement include: providing a sense of purpose, autonomy and control, and social affirmation (Creech et al. 2013), as well as facilitating subjective experiences of pleasure, enhanced social interactions, increased engagement in day-to-day life, fulfilment of musical ambition, and self-satisfaction through musical accomplishment (Perkins and Williamon 2014).

From this growing body of literature, we are able to extract some indication of *why* music may mediate mental health recovery. MacDonald et al. (2012b) suggest that music has many properties that can lead to health benefits, being ubiquitous, emotional, engaging, distracting, physical, ambiguous, social, communicative, and affecting behaviour and identities (pp. 4–6). Clift et al. (2010) discuss what they term ‘generative mechanisms’ identified by choral singers as linking singing with improved wellbeing. These include: engendering happiness and countering feelings of depression, concentration which can prevent worrying, deep breathing which can counteract anxiety, social support which can reduce feelings of isolation, learning which keeps the mind active, and regular commitment that motivates people to remain active (pp. 29–31). In drumming, Newman et al. (2015) demonstrated a raft of mechanisms supporting enhanced wellbeing among carers at a mental health facility: a sense of belonging, relaxation, energy and productivity, learning, enhanced mood, humanising, sense of accomplishment, escape from trauma, and emotional expression (pp. 6–10). Further, Winkelman (2003) argued that drumming can enhance recovery of drug addicts through facilitating relaxation, producing pleasurable experiences, releasing emotional trauma, and allowing for a reintegration of the self through connectedness and spirituality. Finally, Burnard and Dragovic (2014) provide evidence that ‘collaborative creativity’ in a percussion programme enhances wellbeing, allowing for a transformative community in which co-creation, togetherness, making mistakes, and risk-taking are encouraged, and in which a family-like setting facilitates a sense of connection and belonging.

Despite these insights, there is acknowledgment that we know relatively little empirically of *why* music is proving a useful tool in mental health recovery (Creech et al. 2013; Fancourt et al. 2014; Mungas et al. 2014). Indeed, while we demonstrated in previously reported research that group drumming for mental health patients and their carers can enhance wellbeing (Fancourt et al. 2016a, 2016b), these studies stopped short of examining what *features* of the musical intervention enabled this. The present research therefore addresses this gap, investigating the features of group drumming that facilitate mental health recovery.

## Methods

### Participants

Thirty-nine participants took part in the study, as described in Additional file 1 (n = 11 men, n = 28 women). Participants included mental health patients (n = 30), mental health informal and formal carers (n = 6), and participants who identified as both patients and carers (n = 3). Among the participants there were four dyads of patient and carer, who attended the sessions together. Participants were recruited through hospitals, psychologists and psychiatrists working in the UK National Health Service (NHS) or private practice, or through mental health and carer support organisations and charities.

The sample was drawn from a total of 61 mental health patients and carers who engaged in a series of group drumming workshops (see “Procedure” section). We have shown in previous studies that these same participants demonstrated recovery on both psychological and biological levels following group drumming, with significantly increased scores in standardised measures of wellbeing and resilience and significantly lower scores on standardised measures of depression and anxiety as compared with a control group (Fancourt et al. 2016a, 2016b). This article seeks to understand the features of the group drumming intervention that enabled such recovery. The qualitative sample represents 64% of the total drumming group. Selected participants were invited to individual interviews by the research team, chosen according to four parameters: (1) representation of both men and women, (2) representation of patients, formal carers and informal carers, (3) completion of the workshop programme, and (4) willingness to be interviewed. All drummers who completed the workshop programme were invited to the focus group interview and self-selected to attend. The UK NHS National Research Ethics Service approved the project (reference 13/LO/1811), and all participants gave written informed consent prior to the study. No payment was given in exchange for participation.

### Procedure

#### The music intervention

Over the course of one year, four group drumming programmes were provided in West London (UK) over either six or 10 weeks. Drumming has been shown in previous research to elicit positive change in mental health (Faulkner et al. 2012; Wood et al. 2013; Maschi et al. 2013; Venkit et al. 2013) and appears to offer particular scope for community building and connectedness (Burnard and Dragovic 2014; Camilleri 2002; Mackinlay 2014). Furthermore, drumming is a practice that does not require knowledge of musical notation, making it particularly appropriate for a heterogeneous group of participants with varying levels of prior musical engagement. The drumming workshops ran once a week for the duration of each programme and lasted for approximately 90 min, including time for conversation. The workshops were led by a professional facilitator, recruited to join the project through open competition, and supported by three specially-trained student assistants from the Royal College of Music London (RCM). The workshops were designed to be socially and musically inclusive, based upon learning aurally (by ear rather than by notation). The room was set in a circle, with the facilitator and assistants sitting among the participants, and drums were provided each week. A typical workshop consisted of call-and-response exercises and learning drumming patterns that built up into larger pieces, with each group working towards an informal celebratory performance for friends and family at the RCM at the end of the programme. Each group comprised 15–20 members.

#### Methods of data collection

The study design was qualitative, recognising the complex social and musical features of a music-making intervention. Following DeNora and Ansdell (2014), who argue that the links between music and health need to be qualitatively understood ‘from within the situations where it [music] is made, encountered and deployed’, this meant paying close attention to the experiences of participants as they created music together, and prioritising individual perceptions of what it was about group drumming that enabled recovery. Two qualitative methods were employed: semi-structured individual interviews and focus group interviews.

Semi-structured individual interviews were conducted with 11 participants within a week of the completion of each drumming programme. The interview schedule incorporated five main areas, connecting the drumming with both hedonic and eudaimonic definitions of wellbeing: (1) general evaluation of wellbeing; (2) evaluation of the group drumming programme; (3) drumming and feeling well; (4) drumming and functioning well; (5) drumming and recovery. Interviews were conducted in a location and at a time convenient to the participant, lasting approximately 50 min. They were audio recorded with permission and fully transcribed. All were face-to-face interviews, with the exception of one which was conducted over the telephone. The interview schedule is presented in Additional file 2.

To enable a larger number of participants to share their experiences, and to capture the groups’ shared meaning-making, four focus group interviews were also conducted in the week after the completion of each programme. The focus groups were open to all participants who completed the programme, and in total 28 participants attended. The focus group schedule covered the same five areas as the semi-structured interview. Each focus group was facilitated by one member of the research team, comprised on average seven members, and lasted for between 32 and 67 min. The focus groups were audio recorded with permission and fully transcribed. The focus group schedule is presented in Additional file 3.

### Analysis

Analysis of the interview and focus group transcripts was conducted using Interpretative Phenomenological Analysis (IPA), designed to capture the participants’ experiences of group drumming. IPA aims to understand the meanings that an experience holds for participants (Smith et al. 2009) and therefore provided a framework for understanding which features of group drumming were meaningful for those involved, and how they made sense of these meanings in relation to their recovery. IPA has been used effectively in health psychology (Brocki and Weardon 2006) and is employed in several studies exploring the links between music and wellbeing (for example Dingle et al. 2012; Perkins and Williamon 2014). The analysis proceeded in six steps, conducted using the qualitative analysis software NVivo 10. Data from the six-week programme, which ran at an early stage of the project, were analysed first and data from the ten-week programme, which ran at a later stage, were analysed second. First, all transcripts were read multiple times for familiarity before second, emergent meaning units were selected and labelled in NVivo. Third, the meaning units from step two were clustered together to form emergent sub-themes, focusing on any features of the drumming intervention that were reported as aiding recovery. Fourth, the sub-themes were integrated into a table of themes and sub-themes for each individual participant or focus group before, fifth, all individual tables were integrated into one overall table capturing the overarching and sub-themes from the six and ten week data. Lastly, these two overall tables were compared and integrated to develop a final table of themes and sub-themes that represent the identified features of the group drumming. All stages of the analysis were conducted independently by two researchers and cross-checked at each stage to ensure agreement of the final themes and their valid representation of the raw data. Data from the interviews and focus groups were analysed separately but merged as the overarching themes were qualitatively convergent.

## Results

Three overarching themes, supported by 14 sub-themes, emerged from the analysis (see Table 1). Each theme describes the ways in which group drumming was reported to enable recovery, clustered into the specific features of (1) the act of drumming, (2) the group, and (3) the process of learning to drum. These overarching themes were convergent across both patients and carers. In the following, each of the sub-themes is explained and supported by evidence from the dataset.

**Table 1:** Description of overarching themes and sub-themes, summarising the features of the group drumming practice

Theme	Sub-theme	Description
Features of the drumming	1.1 Nonverbal communicating	Drumming as a means for expression and communication without words
	1.2 Rhythmic	Drumming as a shared rhythmic experience, which is primitive and grounding
	1.3 Physical	Drumming as implying bodily effort, energy and release of accumulated tension
Features of the group	2.1 Connecting	Group as constructing a sense of relatedness, unity
	2.2 Belonging	Group as a shared identity, a place of belonging
	2.3 Accepting	Group as accepting, eliciting integration and approval
	2.4 Providing safety	Group as a place of refuge, safety
	2.5 Caring	Group as a place of handing over responsibility, being held by the group
	2.6 Socialising	Group as a means to increase social contact
Features of the learning	3.1 Inclusive	Learning as inclusive, any level of skill welcome, any process adequate
	3.2 No mistakes	Learning as a process in which the concept of fault is dissolved
	3.3 Freeing	Learning as a new experience: no homework, no directedness, no control
	3.4 Embodied	Learning as a process incorporated in the body, a ‘new way of thinking’
	3.5 Role of facilitator	Learning as facilitated by a central, expert musical facilitator

### The specific features of drumming

The first sub-theme centred on the way in which drumming acted as a form of *communication* (sub-theme 1.1):

Entering into the group drumming really is learning a new language (…) there wasn’t anything to say or to internalise or to even judge, it was just about a different way of talking to each other. (Focus group 3, patient).

The ‘different way’ of talking alluded to here was also picked up by another participant, who commented that drumming is ‘another way of expressing or getting out feelings really’ (Celia, patient, interview). Crucially, this form of communication was *nonverbal*:

It’s nonverbal… it just works in a different way you know? Where you don’t have… one doesn’t have to speak just… you can get a benefit out of something that’s not through language… Yeah, it does affect me on some level (…) I can’t always explain my thoughts and feelings but somehow sort of just banging the drum and having that vibration and feeling something just touches something inside, I just… connect with it. (Celia, patient, interview)

This thing [drumming] works on another level. This is nonverbal and using the sounds and the music. (Alison, formal carer, interview)

It would appear that the vibrations (Celia) and sounds (Alison) of drumming are able to elicit a means of communication that is important to these participants *because* it relies on a different communication mechanism than verbal language.

The second sub-theme highlighted the drumming’s *rhythmic features* (sub-theme 1.2):

I would go away and I had them [rhythms] in my head and the ‘boom boom, boribori boom’ and I have them in my head, you know, from my memory, the sound we produced was… amazing. (Focus group 3, patient)

I focus on a sort of driving, repetitive thing that I just liked. (Vicki, informal carer, interview)

As well as the importance of the rhythm in and of itself—as an acoustic outcome of the drumming and as something enjoyable to learn and create within and beyond the workshops—for some participants rhythm also facilitated a connection with life:

I think being in a drumming group was to me like a reconnection with the drumming of everything (…) I think everything that we do, and I think that’s the beauty… the heart, the pulse, it’s just like it’s *there* really and I think it’s in a way it’s kind of reconnect, you know like to me it’s like reconnection with being alive really. (Focus group 3, patient)

Linked to this, the drumming also emerged as a grounding experience, providing security through the experience and ‘feel’ of the beat:

Drumming is very much to do with earth… to do with, with grounding yourself to that, to that wisdom, so yeah… that’s why for me it was very efficient. (Alison, formal carer, interview)

There’s something about the *beat* that’s quite um—it’s sort of like a sort of anchoring process. (Vicki, informal carer, interview)

In short, sub-theme 1.2 evidences the importance of the rhythmical properties of drumming, which could facilitate a connection with the heartbeat, and provided a grounding mechanism through the security of the beat.

Finally, the third sub-theme concerned the *physicality of drumming* (sub-theme 1.3), a process which engaged the body:

It’s probably the most physical activity I do as well… because it´s actually quite physical isn’t it? (John, patient, interview)

There is something very basic about just *hitting* things to make sound, it’s very physical because you can feel the, you can feel your hand resonating when you hit the drum. (Focus group 2, patient)

Recalling that the physical act of playing the drum is also associated with its communicative function (sub-theme 1.1), it also appears linked with the generation of energy and a release in tension:

Even if I felt equally tired, it was sort of a live tiredness rather than a numb, dead, detached tiredness. (Focus group 4, patient)

Just the act of hitting a drum was quite cathartic, I’m not sure it would have worked the same if it was other… like if it was a xylophone. (Elicia, patient, interview)

The drumming, then, emerged as a physical act, implying bodily effort and both the generation, and liberation, of energy.

In sum, this overarching theme has illustrated the specific emergent features of drumming. In particular, drumming materialised as a form of communication, particularly offering an alternative to verbal communication, and as a grounding experience that allowed participants to connect with primal beats and to share a rhythmic experience. Finally, drumming was viewed as a physical act, both generating and liberating energy.

### The specific features of the group

Six sub-themes emerged that characterised specific features of the group, starting with the facilitation of *connectedness* (sub-theme 2.1):

Sometimes the smallest things make such a difference… sometimes we take so many sessions, and…I think it’s the unity you know? Like people sharing something. (Focus group 1, patient)

You know it’s like almost the rest of the group’s like one person. You know it’s like it seems to feel like very cohesive. Like I imagine if you were drumming with one other person, you’d feel you were both a unit and like in the big group you feel like you’re all one unit. (Vicki, informal carer, interview)

These two comments indicate similar but nuanced experiences of connectedness, the first indicating a sense of sharing but the second illuminating a sense of ‘oneness’ through drumming, where individuals become incorporated into a larger group. Indeed, the fact that drumming is *musical* seems central to this point:

Every time I felt more connected after the session. I was like, ‘This doesn’t make much sense, because I’ve not said anything to anyone other than “Hello. How are you?”’(…) And I was like ‘This is quite strange’, because you do sort of connect through that sort of rhythm you’ve built and that *shared* experience. (Focus group 4, patient).

The participants achieved connection through the features of the music itself, in particular forming bonds in and through the rhythmic features of the drumming.

This connectedness also extended to a sense of *belonging* (sub-theme 2.2):

A sense of belonging to something (Imogen, patient and informal carer, interview).

That’s quite, quite nice to feel included in something…you know, because sometimes my illness makes me feel separate from…things you know (Matthew, patient, interview).

Captured in Matthew’s comment is the suggestion that the drumming enabled belonging *despite* the feeling of separation that his illness can elicit. For others, an identity appeared to emerge that was specific to a shared experience of mental health:

That’s what again, the drumming did, I met loads of people and it’s my tribe. (Fiona, patient, interview)

The reason we…why were there in the first place is that each person in the group is there for—we have something in common in the groups. Like my singing for breathing, we all have a condition that requires us to have help with breath and stuff like that, and singing is one way of doing that. So we have that in common. It’s the same thing. (Focus group 4, patient)

The fact that the participants had a shared starting point appeared to act as an enabler to a developing sense of belonging and shared identity.

Third, the group appeared to facilitate *acceptance* (sub-theme 2.3):

Maybe everybody has a different life pattern (…) but when you are coming into the [drumming] circle it’s, you know, you are not bringing in your personal life. You can communicate from where you are at the time (…) So I suppose it’s connecting with people you may not do, who may not even want to connect with you outside, but when you are in that circle then they may get to know you without any views or judgements. (Focus group 3, patient)

The use of the word ‘circle’ appears to capture a protected space in which there is equality, a point picked up by other participants who described integrating into the group in a way that dissolved hierarchies or labels:

Look it was nice not to know who was who yeah? So we didn’t have to put a label ‘this one is a therapist, this one is a patient’. (Alison, formal carer, interview)

It’s a circle… it’s no hierarchy. You find your place in the circle, nobody tells you where to go like in the classroom. (Focus group 1, patient)

The group emerged, then, as facilitating acceptance, with judgements put aside in favour of an inclusive, integrated environment.

Fourth, the group *provided safety* for the participants (sub-theme 2.4):

It gave us a sense—it gave me somewhere to go, a safe space to go each week. (Andrew, Patient, interview)

I must admit you kept it safe for all of us, I did feel safe and that was all credit to everyone and [the facilitator] because he was at the helm and everybody was—they worked their positions, they felt safe enough to explore. (Focus group 3, patient)

The group appeared to act as a place of refuge for some of the participants, providing safety in terms of being part of a shared and accepting space but also a space that ran regularly and reliably. Furthermore, however, the group was seen to be safe musically, allowing for exploration (see also sub-themes 3.2 and 3.3). Linked with this point, sub-theme 2.5 highlights the ways in which participants experienced the group as *caring*:

I didn’t feel that I had to sort of care for her in that one hour, that hour and a half space. (Vicki, informal carer, interview)

There was a student there that, you know, she was like I thought she was very perceptive and trying to sort of (giggle) help because I wasn’t playing the… drum… I don’t think I could get the drum beat…so I found one of the students sort of really sweet and you know, yeah trying to help at the side and I did ask her once yeah if she could sit at the side because that was helpful. (Celia, patient, interview)

Importantly, the group seemed to facilitate care in different ways for different participants, depending on their individual needs.

The final sub-theme to emerge concerns the *socialising* that the group enabled (sub-theme 2.6):

We were all encouraged to meet each other afterwards. That was very much a part of this as well. (Fiona, Patient, interview)

The group acted as a source of new social contact, with participants regularly meeting before and after the sessions in the café attached to the venue.

In sum, this overarching theme has illustrated some of the specific features of the group practice. In particular, the group emerged as a space where participants found acceptance, and where judgements were put aside in favour of an inclusive, integrated environment. The group provided a means for the participants to achieve connection through the features of the music itself, becoming connected in and through the rhythmic features of the drumming, and developing a sense of group identity. Finally, the group provided a place of safety or refuge, in which participants felt that they were taken care of, as well as facilitating new social interactions.

### The specific features of the learning

Five sub-themes emerged concerning the specific mechanisms of learning experienced within the musical group practice. First, learning appeared in this context to be highly *inclusive* (sub-theme 3.1):

I like the way it didn’t matter if you were getting it, or you don’t have to do all the beats, and you can do the ones that you remember (…) you’re not feeling left out and feeling like you’re a failure. So afterwards you’d feel, “Oh! I did drumming”. (Focus group 4, patient)

There’s another language in the room which *everyone* could just pick up and *talk* with. That’s got to be the immediate attraction to it, that ‘oh, okay, well, I’m not you know so great at it at first, but I can—I’m picking up’ and you see yourself instantly picking it up. (Fiona, patient, interview)

The key points here concern, first, the attitude to learning constructed within the group. This was built around an environment in which everyone’s contribution was equally valued and, crucially, participants could come in and out of the drumming at their own pace without experiencing feelings of ‘failure’. Central to this process, second, was the drumming itself, which allowed everybody to make a musical contribution immediately, regardless of their previous musical experiences.

Closely linked with this, the participants also identified a change in the way that they thought about, and dealt with, *mistakes* (sub-theme 3.2):

I was quite nervous and it was really important to me the language that was used, which was ‘make as many mistakes as you like’ you know and ‘if you make a mistake, make sure I hear it’ and it was all… such a forgiving atmosphere… I found that really encouraging and all my worries just left me… It takes away the framework of failure, disappointment and shame (…) and when you take that framework, you have people that are not being graded, you’re not performing to anybody, you’re just speaking… when you take all that off then you get this liberty. (Focus group 1, patient)

Here, we appear to see the participant reframing how they think about participation, recognising that musical engagement can be free from failure or shame. For people suffering from mental distress, such realisation may be crucial to facilitating further participation in activities that may support recovery.

Moving to the third sub-theme, the drumming sessions introduced an element of *freedom* into learning (sub-theme 3.3):

I felt the teaching style was quite different from anything that I’d experienced before. I have done a bit of music and a bit of drumming as well, before, but it’s always been about counting and writing it down. (Focus group 4, patient)

I felt I had permission to do my own thing within the group. (Andrew, patient, interview)

Freedom here emerges in two different ways, either as a new form of musical learning that moves away from more formal models relying on written musical notation, or as a sense of agency within the group itself.

Further, the participants described encountering a new type of *embodied* learning (sub-theme 3.4):

What I started to notice (…) was that your *body* remembers it, certain patterns and certain things (…) and you’ve learned it and you can’t unlearn it. (Imogen, patient and informal carer, interview)

It involved thinking, but it involved thinking in a different way…it wasn’t intellectual thinking. It was just *feeling* thinking if that’s a term. (Andrew, patient, interview)

Learning the drum, then, often seemed to be about ‘unlearning’ old learning processes based on cognition, and allowing and trusting the body to internalise and reproduce the beats. In this process, there also appeared to be a certain degree of ‘letting go’ of thoughts and analysis, in favour of trusting the body to learn and remember.

The final sub-theme in this category recognises the *central role of the facilitator* (sub-theme 3.5):

It’s about the teaching. It’s about energy and the chemistry between you and that person. He did an amazing job. (Fiona, patient, interview)

Just the way the teacher was, that helped, a lot. Um, how he was with people and passionate about the drum. (Celia, patient, interview)

Underpinning many of the above sub-themes, then, seems to be the connection formed between the facilitator and the participants, based on humour, inclusion, energy, passion, and the dissolution of fault.

In sum, this overarching theme has illustrated specific features of the learning process. In particular, learning was framed as an inclusive activity, open to everyone, in which mistakes were welcomed and reframed as part of the musical practice. Within the direction provided by the facilitator there was also freedom for each individual to contribute musically, and learning emerged as an embodied process. Finally, the role of the facilitator transpired as central to all of the above, underpinning and supporting the learning process and ethos.

## Discussion and conclusion

This research has revealed three overarching features of a group drumming intervention known to enhance recovery (Fancourt et al. 2016a, 2016b): (1) the specific features of drumming, including drumming as a form of non-verbal communication, as a connection with life through rhythm, and as a grounding experience that both generates and liberates energy; (2) the specific features of the group, including the group as a space of connection in and through the rhythmic features of the drumming, as well as facilitating feelings of belonging, acceptance, safety and care, and new social interactions; (3) the specific features of the learning, including learning as an inclusive activity in which the concept of mistakes is dissolved and in which there is musical freedom, supported by an embodied learning process expedited by the musical facilitator. Building on the growing body of literature demonstrating the impact of the arts, and music in particular, on wellbeing (The RSPH Working Group on Arts, Health and Wellbeing 2013), this study contributes insight into the mechanisms *behind* the impacts, illuminating the specific features of a group music practice. In so doing, it lends support to DeNora and Ansdell’s (2014) critique that experimental procedures alone cannot illuminate the processes of change elicited by music: ‘that a slower form of dwelling with music in situ can help us to see the variegated processes by which music helps’.

Revisiting our conceptual starting point of mental health *recovery*, the evidence presented here lends support to Crawford et al.’s (2013) extended notion of *creative practice as mutual recovery.* The ‘mutual’ in ‘mutual recovery’ positions the building of communities and social relationships as integral to mental health recovery, providing spaces of ‘mutual hope, compassion and solidarity’ (p. 58). Crucially, this mutuality can be both within and between different groups of people, opening the possibility for shared experiences and relationships to support recovery among hospital patients recovering from critical illness (Chiang 2011), among peers recovering from mental illness (Repper and Carter 2011), or among mental health nurses and their patients (Hostick and Mcclelland 2002). ‘Creative practice’ refers to the above-cited point that the arts may provide new ways of enabling such recovery though providing forums for shared understanding and mutuality.

Indeed, the findings of this study—alongside previously reported evidence that the group drumming programme under investigation led to enhanced mental health (Fancourt et al. 2016a, 2016b)—illuminate the specific ways in which a *creative practice* can mediate recovery, highlighting features that are highly specific to music and, in this case, to drumming. Echoing Nettl’s (2015) definition of ‘music as human sound communication outside the scope of spoken language’ (p. 28), the ability of drumming to facilitate nonverbal communication appears an important feature in its potential for facilitating recovery. Particularly for the participants in this study, the opportunity to communicate without needing to describe emotions, feelings or thoughts in words, or even to talk with other members of the group, appeared fundamental as a mechanism for creating a forum for expression and connectedness *through* the relatively safe medium of drumming. Indeed, it appeared that the ‘words’ of verbal language were replaced by the beats and rhythms of the drumming, which were experienced both in sound as well as in the body, facilitating what participants described as a grounding and primitive source of connection. Furthermore, and linking with other literature supporting the idea that physical activity can benefit positive mental health (NEF 2011; Richardson et al. 2005), the drumming enabled a physical experience, generating and liberating energy in a way that allowed tension to be released and physical tiredness to replace, or alleviate, mental tiredness.

These musical mechanisms do not stand in isolation, however, being closely linked with the *mutuality* of the practice. Indeed, the specific mechanisms of the drumming *need* to be understood alongside the mechanisms of the group, which—largely through the rhythmic features of the drumming—facilitated strong connections as well as a space of acceptance and safety. This *mutual* experience, constructed within each group, in which hierarchies were removed and shared identities built, appeared central to allowing all members to find meaning and recovery in the activity. As Crawford et al. (2013) point out, the ‘relational ontology of recovery is important’ (p. 57), foregrounding the *shared* nature of the recovery practice. In this project, this meant bringing together patients, carers, and musicians in the same room and removing labels, roles, or hierarchies in order to share and co-construct ‘interactional processes, based in social relationships and situational identities’ (p. 57) with the aim of achieving recovery for all. That the same themes were emergent across participants highlights the importance of this finding, with patients and carers often sharing the experiences that led to recovery. The mechanisms of recovery thus become understood as social mechanisms, constructed in and through the mutual practice of drumming.

**Fig. 1:**
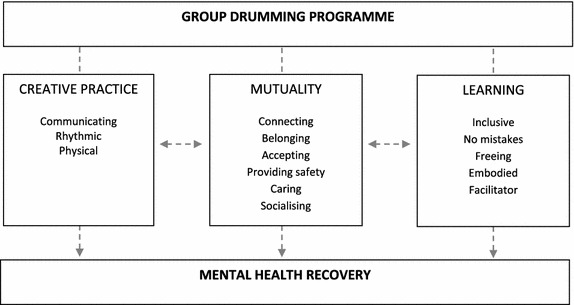
Evidence for creative practice, mutuality and learning as mechanisms in mental health recovery

Further, a third mechanism, concerning the specific mechanisms of *learning* a musical practice, emerged that connects together the act of playing the drum and of doing so within a group. Echoing one of the New Economic Foundation’s (2011) *Five Ways to Wellbeing* and picking up on previous studies documenting the role of musical accomplishment in enhancing wellbeing (Newman, Maggott, and Alexander 2015; Perkins and Williamon 2014), learning something new appeared important in the recovery process in and of itself, but the *way* of learning also appeared crucial. In line with Burnard and Dragovic (2014), making mistakes and risk-taking were key to the constructed learning ethos, reframing music as an activity that facilitates freedom and that is learned through the body, relieving more ‘traditional’ notions of learning as a directed and cognitive activity. The drumming space was one where one could not go ‘wrong’, and where there was a collective learning community but also the freedom to explore musically and personally. Both for participants new to drumming and those with previous musical experience, these learning mechanisms were central to the potential for recovery; they form what Smilde et al. (2014) refer to in their work on music and dementia as a ‘space of social learning’ (p. 246). Within this space, recovery mechanisms appear to manifest at the intersection of the drumming, the group, and the process of learning, as represented in Fig. 1. Group drumming can be said to provide a creative and mutual learning space in which mental health recovery can take place.

While shedding light on the mechanisms of mutual recovery in group drumming, there remains further conceptual and empirical work to be done to further unpack and evidence the notion of *creative practice as mutual recovery* (Crawford et al. 2013). The qualitative design means that the mechanisms reported here are, by necessity, specific to the four drumming programmes included in the research. While we needed to examine these programmes in detail in order to extract the ways in which the practice contributed to recovery, this means that the resulting mechanisms cannot be generalised to other drumming practices. Indeed, it may well be that several of the mechanisms arose as a function of the larger aims of the project and the design of the programme with the aim of recovery in mind; the sessions were intended to be as welcoming, inclusive, and musically and personally safe as possible. We certainly cannot assume that other drumming or creative practices would have similar features, though the findings do resonate with other similar studies in the field (Burnard and Dragovic 2014; Newman et al. 2015; Winkelman 2003). Additional research is needed to ascertain just how project-specific the findings are, and whether similar features of drumming, group, and learning would underpin the impact of creative practice on recovery in other contexts. Furthermore, the extent to which recovery is and can be mutual requires further exploration, to more fully illuminate the complex social relationships, including issues of power, that are intertwined within creative practice.

Finally, this article focuses only on the features of group drumming that facilitate mental health recovery, and does not consider other explanations for the recovery elicited by this intervention. Our previous research, however, provides exploratory evidence that drumming could modulate similar biological pathways to other psychological interventions (Fancourt et al. 2016a, 2016b). Indeed, Fancourt et al. (2014, p. 24), in their systematic review of the psychoneuroimmunological effects of music, argue that future research investigating the impact of music-making on health should: (1) provide clear descriptions of the types and length of stress experienced by participants, (2) give clear descriptions of the aural, physical, social and personal perception of the music involved, an aim met in the current research, (3) consider groups of biomarkers in conjunction with one another, and (4) test models of the psychological, neurological and immunological mechanisms behind effects. While the field remains some way from achieving such ideals, an interdisciplinary approach to the study of music and recovery is indeed likely to elicit further understanding of the complex mechanisms behind music’s impact on recovery.

Notwithstanding the limitations cited above, this study offers a timely contribution to the literature, generating insight into the specific features of a music-making intervention known to facilitate recovery. The three features outlined in this article go some way to addressing the ‘how’ in Staricoff’s (2006) argument that ‘the value of evaluating the effect of the arts in healthcare resides in providing to all involved in designing, implementation and funding, the knowledge of what, when and how to introduce different art forms to achieve the most effective results’ (p. 116). Implications for practice include the importance of recognising the communicative function of music, particularly in its nonverbal form, as well as the central role played by rhythm and beat, both aurally and through the physical connection with the drum. Further, the mutual aspects of the practice point towards the value of group activities, carefully fostered in order to maximise opportunities for acceptance, identity building, and connectedness, within a learning environment that removes the fear of mistakes and that embraces an embodied, rather than cognitive, approach to music-making. Finally, as evidenced in this research, the choice of musical facilitator is crucial, acting as a conduit for recovery through his or her input and ability in fostering the mechanisms outlined in this study. Further opportunities for musicians to experience and train in facilitating music practices for mental health recovery, including their own, are certainly warranted.

## Additional files

**Additional file 1.** Participants.

**Additional file 2.** Semi-structured interview schedule.

**Additional file 3.** Focus group schedule.
