# Supplementary material for: Making music for mental health: how group drumming mediates recovery
Source: Psychol Well Being. 2016 Nov 29;6(1):11. doi: 10.1186/s13612-016-0048-0 (PMC5127870; doi:10.1186/s13612-016-0048-0)
Supplement: Supplementary file 1 — Additional file 1. Participants. [file 13612_2016_48_MOESM1_ESM.docx]

**Additional File 1. Participants.**

| Participant | Sex | Data | Programme |
| --- | --- | --- | --- |
| Patient [John]* | M | Individual interviews | 6-weeks |
| Carer informal [Jane] | F |  |  |
| Carer formal/Patient [Louisa] | F |  |  |
| Carer formal [Alison] | F |  |  |
| Patient [Elicia] | F |  |  |
| Patient | F | Focus group 1 |  |
| Patient | F |  |  |
| Patient | F |  |  |
| Patient | F |  |  |
| Carer formal | F |  |  |
| Patient | F |  |  |
| Carer formal | F |  |  |
| Carer formal | M |  |  |
| Patient | M |  |  |
| Patient | M |  |  |
| Patient/Carer informal [Imogen] | F | Individual interviews | 10-weeks |
| Patient [Celia] | F |  |  |
| Patient [Matthew] | M |  |  |
| Patient [Andrew] | M |  |  |
| Patient [Fiona] | F |  |  |
| Carer informal [Vicki] | F |  |  |
| Patient | F | Focus group 2 |  |
| Patient | M |  |  |
| Patient | F |  |  |
| Patient | M |  |  |
| Patient | F |  |  |
| Patient | M |  |  |
| Patient | M |  |  |
| Patient | F |  |  |
| Patient | F |  |  |
| Patient | F |  |  |
| Patient/Carer informal | F | Focus group 3 |  |
| Patient | F |  |  |
| Patient | F |  |  |
| Patient | F |  |  |
| Patient | M |  |  |
| Patient | F |  |  |
| Patient | F | Focus group 4 |  |
| Patient | F |  |  |

**Pseudonyms are provided for participants interviewed individually. Focus group participants are identified by the number of the focus group that they attended.*
