# Supplementary material for: Making music for mental health: how group drumming mediates recovery
Source: Psychol Well Being. 2016 Nov 29;6(1):11. doi: 10.1186/s13612-016-0048-0 (PMC5127870; doi:10.1186/s13612-016-0048-0)
Supplement: Supplementary file 2 — Additional file 2. Semi-structured interview schedule. [file 13612_2016_48_MOESM2_ESM.docx]

**Additional file 2. Semi-structured interview schedule.**

| **Topic** | | | **Indicative questions** |
| --- | --- | --- | --- |
| General wellbeing evaluation | | | How have you been feeling?  How would you grade your general wellbeing?  In the past few weeks how often have you felt happy? |
| Evaluation of programme | | | How did you feel when you started the programme? How do you feel now? |
|  |  |  | Tell me about your experiences in the sessions?  What were the greatest moments?  What, if any, were the challenges of this programme?  Suppose that you were in charge and could make one change that would make the program better. What would you do? |
| Feeling Well | | Positive Emotions | “During the past few weeks, how often did you feel…  … happy?  …interested in life?  …satisfied with life?” |
|  |  | Engagement | What were the most enjoyable tasks during the sessions?  How often have you felt completely immersed in the tasks of the sessions?  Were there any tasks with which you found yourself loosing track of time? How often did this happen?  How often did you feel particularly excited with the sessions? |
| Functioning well | Socially | Personal Relationships | Let´s talk a little about relationships. In the last few weeks, how often did you feel that you had warm and trusting relationships?  How satisfied are you with your personal relationships? |
|  |  | Social Adjustment | How was the working in a group experience?”  Have you noticed any difference in your interactions with others since the programme started? |
|  | Personally | Self-concept | Imagine you had to describe yourself to someone that just met you, what would you say? |
|  |  | Meaning | During the programme, how often did you feel that you had experiences that challenged you to grow positively?  Have you noticed any difference in the way you think about life recently?  In general, to what extent do you feel what you do in your life is valuable and worthwhile?  In the past month, how often did you feel that your life has a sense of direction and meaning to it?  To what extent do you feel this programme was worthwhile? |
|  |  | Accomplishment | What do you feel you have gained from these sessions?  In the past month, how often did you feel good at managing the responsibilities of daily life? |
| Recovery | | | How do you think this has helped you deal with (condition)?  What do you think it is about the sessions that help in that?  How was the experience of having people from so many different backgrounds here? |
| Overall wellbeing | | | Taking all things together, how happy would you say you are these days? |
