# Supplementary material for: Making music for mental health: how group drumming mediates recovery
Source: Psychol Well Being. 2016 Nov 29;6(1):11. doi: 10.1186/s13612-016-0048-0 (PMC5127870; doi:10.1186/s13612-016-0048-0)
Supplement: Supplementary file 3 — Additional file 3. Focus group schedule. [file 13612_2016_48_MOESM3_ESM.docx]

**Additional file 3. Focus group schedule.**

| **Introduction** | Welcoming participants; introductions  Explaining purpose and context of the focus group (no right or wrong answers, keeping confidentiality within the group)  Explaining about the research project and ethics, that information is confidential and no names will be used |
| --- | --- |
| **Main body** | General wellbeing evaluation: How would you rate your wellbeing these days?  Evaluation of the programme: What is your general evaluation of the experience?  Hedonic and Eudaimonic wellbeing: How did this programme affect the way we feel day-to-day? How did you experience doing music as part of this particular group?  Recovery: How does the making of music help us in this change (if evidenced)?  Transition: Of all the things we discussed, what do you think is the most important aspect to take from this experience? |
| **Close** | Thanks; contact information for further follow up; explain how data will be used |
